# Intrauterine device embedded in omentum of postpartum patient with a markedly retroverted uterus: a case report

**DOI:** 10.1186/s13256-017-1480-3

**Published:** 2017-10-25

**Authors:** Dana A. Neumann, Joseph A. Graversen, Suzanne K. Pugh

**Affiliations:** 1 Philadelphia College of Osteopathic Medicine, 4170 City Ave, Philadelphia, PA 19131 USA; 2 Paoli Hospital, 255 West Lancaster Ave, Paoli, PA 19301 USA

**Keywords:** Intrauterine device, Uterine perforation, Risk factors, Uterine position, Postpartum, Breastfeeding

## Abstract

**Background:**

The intrauterine device is a popular form of long-acting reversible contraception. Although generally safe, one of the most serious complications of intrauterine device use is uterine perforation. Risk factors for perforation include position of the uterus, force exerted during intrauterine device insertion, postpartum period, and breastfeeding. This case is important and needs to be reported because it highlights the need to assess risk factors for uterine perforation. It adds to the medical literature because it examines the relationship between position of the uterus and the location of uterine perforation. This case report is unusual in that it describes the mechanism and specific location of uterine perforation in relation to the position of the uterus.

**Case presentation:**

We present a case of an intrauterine device found in the omentum of a 30-year-old white postpartum woman with a significantly retroverted uterus after the intrauterine device threads were not visualized on speculum examination during a 6-week placement check. The intrauterine device was located and removed via laparoscopy without complication.

**Conclusions:**

This case report will be of interest to women’s health practitioners because it illustrates the importance of identifying patients with risk factors for uterine perforation, examining the relationship between uterine position and location of perforation. This is especially significant because the true incidence of perforation may be higher than the numbers reported in the literature. There is no specific diagnostic code for uterine perforation and it is unlikely that retrospective studies can accurately identify all cases.

## Background

The intrauterine device (IUD) is a popular, long-acting reversible contraceptive (LARC) device. Since 2002, IUDs have accounted for the largest proportion of LARCs. The use of IUDs has increased from 3.5% in 2006 to 2010 to 6.4% in 2011 to 2014 [1]. The benefits of an IUD include technically simple insertion, reversibility, extended duration of use, and high efficacy [2, 3]. However, IUD use is not without risks. One of the most serious complications is uterine perforation and migration of the device to the pelvic or intra-abdominal cavity. The reported incidence of IUD perforation of the uterus ranges from 0.2 to 9.6 per 1000 insertions [4]. Previous cases reports noted uterine perforations in which the IUD migrated to the free peritoneal cavity, adnexa, omentum, beneath the vesicouterine peritoneum, within the broad ligaments, small intestine, and bladder. Factors that increase the risk of perforation include flexion of the uterus, force exerted during IUD insertion, and rigidity of the IUD and inserter [5]. Other risk factors include the postpartum period and breastfeeding [6]. We report an unusual case of a perforated IUD embedded in the omentum of a postpartum patient with a markedly retroverted uterus.

## Case presentation

**Fig. 1:**
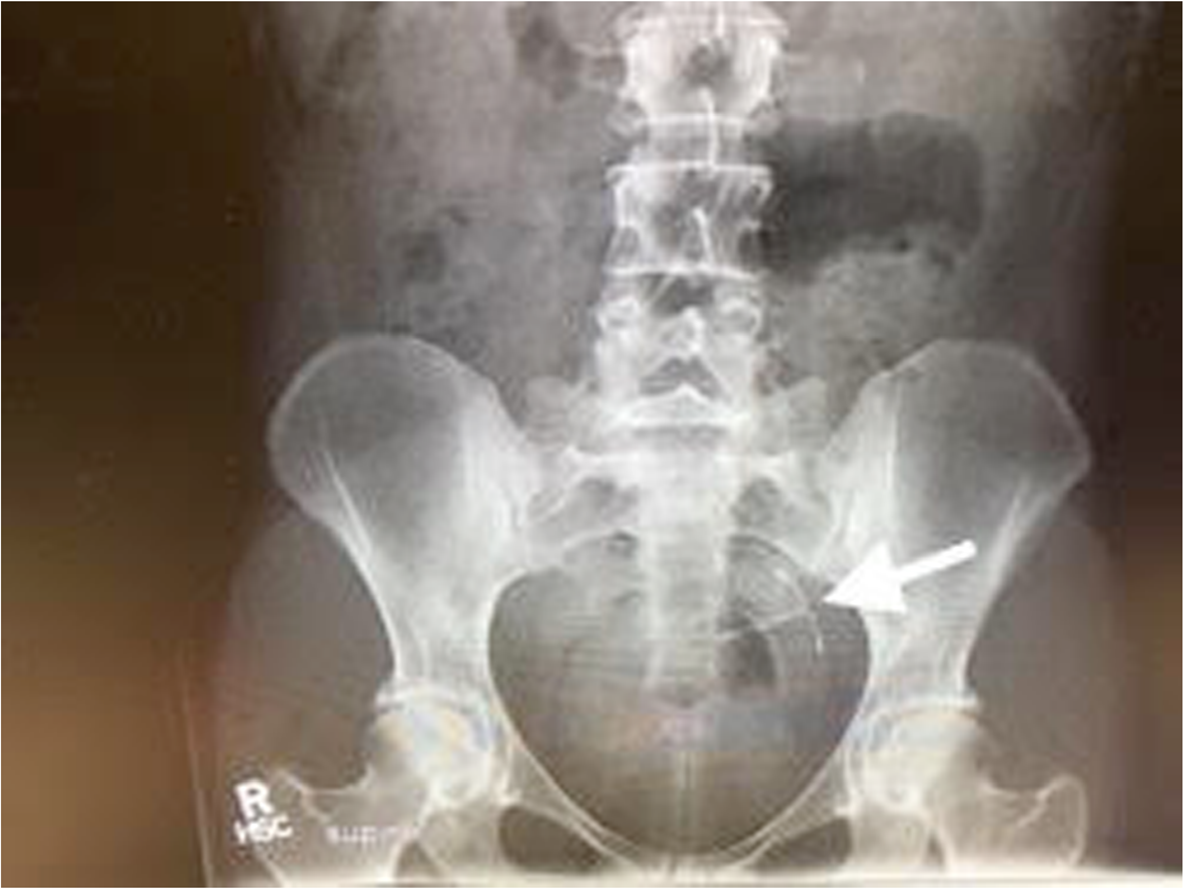
Abdominal radiograph of pelvis with arrow pointing to arms of intrauterine device

A 30-year-old white woman, gravida 1, para 1, presented to our hospital for scheduled laparoscopy after IUD threads were not visualized on speculum examination during a 6-week placement check. The hormonal IUD had been placed 8 weeks after an uncomplicated cesarean delivery and our patient was concurrently breastfeeding. Pelvic ultrasonography did not show intrauterine placement of the IUD. Subsequent abdominal radiography located the IUD in the left anterior aspect of her pelvis (Fig. 1).

**Fig. 2:**
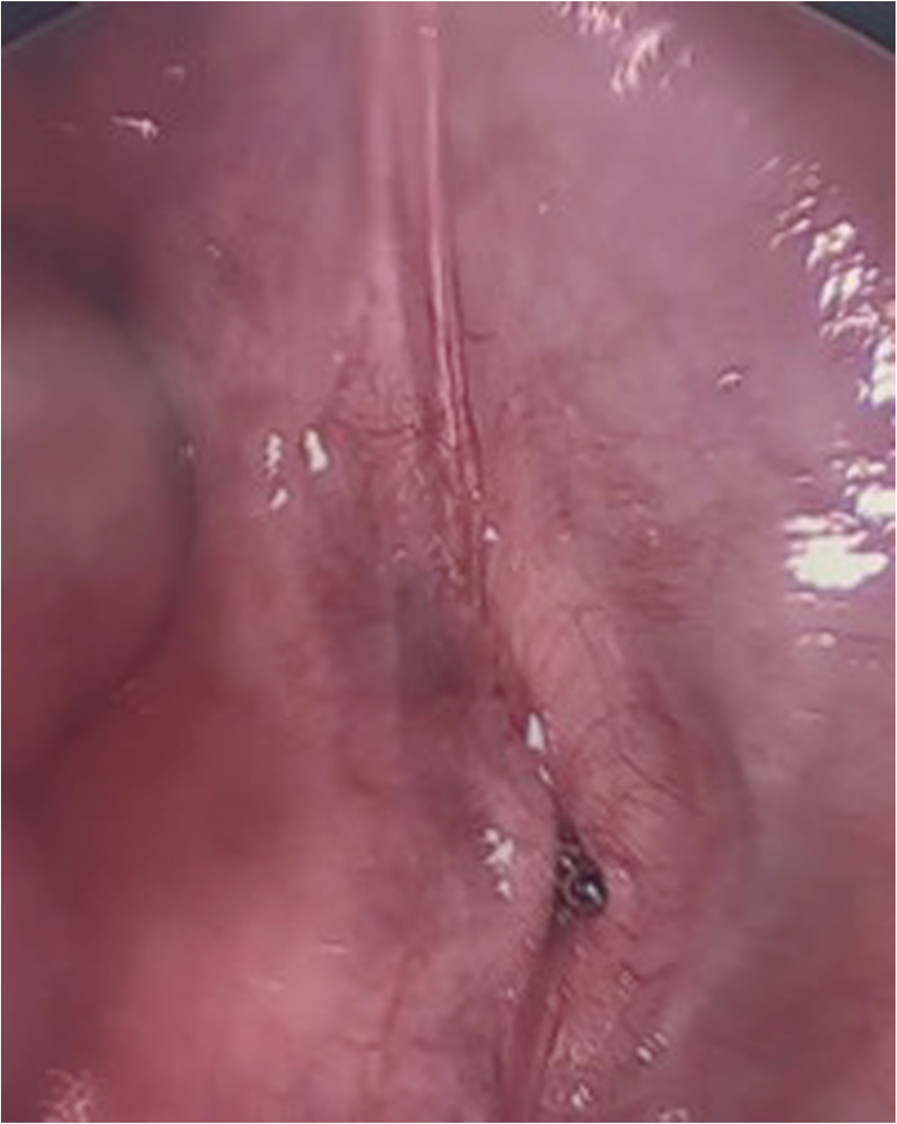
Intraoperative view of uterine manipulator perforating peritoneal cavity at junction of cervix and bladder

She was scheduled for a laparoscopy to remove the IUD. An acorn uterine manipulator was used to assist with visualization, elevation, and movement of her uterus and adnexa during the procedure. It was easily placed into the endocervical canal without complication. A 5 mm infraumbilical incision was made for laparoscopic port placement. A laparoscope was passed through the port to visualize the intraperitoneal cavity. Her fallopian tubes and ovaries appeared normal. A normal appearing, but markedly retroverted uterus was also noted. Laparoscopic exploration of the peritoneal space revealed that the IUD was not in her pelvis or anterior cul-de-sac as originally suspected. Due to retroflexion of the uterus, the uterine manipulator was then used to elevate her uterus. When this was done, the stem of the tip of the uterine manipulator was visualized having perforated the peritoneal cavity at the junction of her cervix and her bladder (Fig. 2). Due to the proximity of the perforation to our patient’s bladder, there was concern for possible bladder injury. A Foley catheter was placed and her bladder subsequently distended and distension was maintained. The uterine manipulator was removed, and the area of perforation was noted to be hemostatic. Her bladder was once again filled and adequate distension was visualized.

**Fig. 3:**
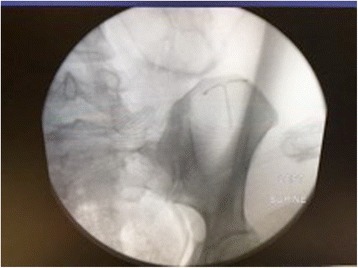
Intraoperative kidney-ureter-bladder X-ray with fluoroscopic guidance showing intrauterine device anterior to patient’s left acetabulum

An intraoperative abdominal kidney-ureter-bladder X-ray (KUB) with fluoroscopic guidance located the IUD anterior to our patient’s left acetabulum, a more superior and lateral position than in the previous abdominal film (Fig. 3). The IUD was located in the omentum of her left mid-abdomen using the laparoscope. The IUD was then removed without complication.

Urology was consulted and performed intraoperative cystourethroscopy which revealed no bladder, urethral, or ureteral injury and no evidence of bleeding. Careful laparoscopic visual inspection of intra-abdominal contents revealed no injury to bowel or vasculature. A speculum examination at the conclusion of the procedure revealed a normal appearing cervix. Her postoperative recovery was uneventful.

## Discussion

The ease in which the tip of the uterine manipulator perforated the junction of the cervix and the bladder into the peritoneal space and the lack of bleeding at this site intraoperatively suggests that there was previous trauma to this area. In a literature review, Zakin *et al*. [5] noted that in 92 cases studied, 18 perforations occurred in the lower uterine segment and upper cervical canal, not infrequently under the vesicouterine peritoneum. We postulate that the IUD originally perforated at this location at the time of insertion. Various authors discuss that perforation caused by an IUD most often occurs during insertion [5, 7, 8], and that it may be secondary to an incomplete perforation that occurs when the uterine wall is damaged and weakened by the metal sound [7].

Insertion during the postpartum period [6, 7] and concurrent lactation [6, 9] are known risk factors for IUD perforation. Andersson *et al*. [10] found that 90% of women with IUD perforations had the IUD inserted within 1 year after pregnancy, with 62% of these patients having the IUD inserted within 12 weeks of delivery. Caliskan *et al*. [6] reported similar findings, that 87.3% of women with IUD perforations had the IUD inserted within 1 year of pregnancy, with 47.2% of these patients having the IUD inserted within 12 weeks of delivery. Postpartum estrogen levels are low during breast feeding, causing the uterus to consistently decrease in size and increase the risk for perforation [10]. Oxytocin production during lactation may also contribute to uterine contractility and involution [9]. Furthermore, increased levels of endorphins in lactating women may increase the risk of undetected perforation because decreased pain may allow the perforation to go unnoticed [10]. Of note, history of caesarean section has been found to not be a risk factor for uterine perforation [11].

Although a controversial topic in the reviewed literature, we felt that uterine position and the risk of IUD perforation was worth exploring. A large study (*n* = 5520) by Chi *et al*. [12] sought to determine whether uterine position affects the risk of IUD perforation and found that women with retroverted uteri did not have a higher incidence of insertion failure or uterine perforation when compared to the anteverted or mid-positioned group. Other authors disagreed, claiming that sharp uterine retroflexion is considered a risk factor for IUD perforation [3, 7, 8]. In this case, we feel that the retroverted nature of our patient’s uterus played a significant role in the perforation of the uterus during IUD placement. Given the hemostatic nature of the wound and the final location of the IUD within the abdominal cavity, we assume that the tract of perforation made during placement allowed the uterine manipulator to easily follow the same path.

It should be noted, however, that there no known studies at this time that explore whether a retroverted uterus is more likely to lead to perforation at the junction of the cervix and the bladder. The mechanism and specific location of uterine perforation in relation to the position of the uterus is not always well described in the literature. This certainly impacts whether uterine position is predictive.

## Conclusions

This case highlights that although perforation is a rare complication of IUD use, it is important to consider the above risk factors when choosing an IUD as a method of contraception. In particular, appropriate selection of contraception is important when a patient presents with multiple risk factors for uterine perforation. This case adds to the medical literature because it examines the relationship between position of the uterus and the specific location of uterine perforation. It is also important to note that the true incidence of IUD perforations may be higher than the numbers reported in the literature. Although there is a diagnostic code for uterine injuries, there is no specific diagnostic code for uterine perforation and it is unlikely that retrospective studies can accurately identify all cases [13].

## Abbreviations

IUD: Intrauterine device
LARC: Long-acting reversible contraceptive
